# A simple and standardized method supports efficient derivation of clinical-grade human embryonic stem cells under feeder- and xeno-free conditions

**DOI:** 10.1186/s13287-025-04831-3

**Published:** 2025-12-01

**Authors:** Qiong Zhang, Huicheng Chen, Jintao Cun, Qianqian Liu, Hao Wang, Luxi Jiang, Shubo Hu, Hongkun Wang, Jingxin Wang, Jian Xu, Jinyue Shi, Fangyuan Sun, Xianlang Xiong, Zhen Liang, Yuansong Yu, Yuanyuan Du

**Affiliations:** 1 Obstetrics and Reproductive Medicine Center, Affiliated Hospital of Yunnan University, Kunming, Yunnan China; 2 Kunming City Maternal and Child Health Hospital, Kunming, Yunnan China; 3 Hangzhou Institute of Medicine (HIM), Chinese Academy of Sciences, Hangzhou, Zhejiang China; 4 Assisted Reproduction Unit, Department of Obstetrics and Gynecology, Sir Run Run Shaw Hospital, School of Medicine, Zhejiang University, Hangzhou, China; 5 Dental Stem cell Bank and Research Center, Savaid Stomatology School, Hangzhou Dental Hospital, Hangzhou Medical College, Hangzhou, China; 6 Hangzhou Miracle Biotech Co., Ltd, Hangzhou, China

**Keywords:** Human embryonic stem cells, Clinical-grade, Derivation under feeder- and xeno-free conditions

## Abstract

**Background:**

Human embryonic stem cells (hESCs), as naturally pluripotent stem cells, constitute a pivotal cell source for cell replacement therapies. Yet, the generation of clinically compliant hESC lines under feeder-free and xeno-free conditions remains inefficient. Moreover, the derivation of hESCs from clinically surplus and discarded low-quality embryos using the standardized, translation-ready culture systems has not been reported.

**Methods:**

By optimizing culture conditions and inner cell mass (ICM) isolation method, we developed a method that significantly improves the derivation efficiency of hESC lines from clinically surplus and discarded frozen-thawed embryos under feeder- and xeno-free conditions. The derivation protocol is operationally simple and easily standardized, with the reagents commercially available and of GMP-grade.

**Results:**

Using this protocol, we successfully established 16 hESC lines. Among blastocysts with morphologically distinct ICMs of grades A and B, the derivation efficiency achieved approximately 60%, with all three grade A ICMs yielding viable hESC lines (100% derivation efficiency for grade A). Notably, for embryos with poorly developed ICMs (grade C), the derivation efficiency of hESC lines approached 30%, showing the protocol’s robustness across varying ICM quality. Adhering to GMP standards, we derived two clinical-grade hESC lines, which were demonstrated biological safety, sustained pluripotency, and the capacity for three-germ-layer differentiation.

**Conclusions:**

Our study offers a robust, standardized, and simple method for deriving clinical-grade hESCs. Efficient derivation, propagation and banking of hESC lines from frozen-thawed embryos would offer a valuable cell source for advancing regenerative medicine, disease modeling, and drug development.

**Supplementary Information:**

The online version contains supplementary material available at 10.1186/s13287-025-04831-3.

## Background

Human pluripotent stem cells (hPSCs) can self-renew indefinitely and differentiate into any cell type in the body, making them highly versatile for developing therapies to treat various diseases [1, 2]. hESCs, a type of hPSCs, are directly derived from early-stage embryos. These embryos have not undergone extensive cell division or exposure to various environmental factors, resulting in minimal accumulation of somatic mutations and manifestation of aging-related phenotypes [3, 4]. Furthermore, as naturally pluripotent cells, hESCs do not require complex reprogramming to achieve pluripotency. Thus, they carry no risk of introducing genetic or epigenetic aberrations associated with somatic cell reprogramming [5–8]. Therefore, hESCs are considered a promising cell source for generating various human cells for clinical applications.

Previous studies have commonly used human feeder layers to establish clinical-grade hESC lines [9–13]. However, feeder cell-based systems can vary in quality depending on the preparation and source of feeder cells, leading to batch-to-batch variability. Feeder- and xeno-free conditions provide more consistent and standardized culture environment, which are critical for reliability and reproducibility, the key requirements for translating stem cell-based therapies to clinical settings. Recent progress has been made that using defined extracellular matrices to enable the xeno-free derivation of hESCs [14, 15]. However, the efficiency of hESC derivation was low. Furthermore, the derivation of hESCs from low-quality embryos under feeder- and xeno-free conditions remains unreported.

Here we report a protocol to effectively derivation of hESC lines under feeder- and xeno-free conditions, with an average derivation efficiency of 45.7%. Notably, for blastocysts at 5-day post-fertilization with visible ICMs (grades A and B), the efficiency exceeded 70%, resulting in the derivation of 8 hESC lines from 11 embryos. For low-quality embryos lacking visible ICMs (grade C), the derivation efficiency was approximately 30%. The established hESCs could subsequently be propagated and cryopreserved under xeno-free conditions. By rigorous screening eligible donors and adherence to Good Manufacturing Practice (GMP) standards, we generated two clinical-grade hESC lines using this protocol, which could be suitable for future transplantation therapies.

## Methods

### Embryo thawing

This study was approved by the Medical Ethics Committee of Affiliated Hospital of Yunnan University (Ethical No. 2023224). All embryos used in this study were donated by couples who provided informed ethical consent. For embryo thawing, the commercial thawing media kit (KITAZATO, VT102) was used. The detailed protocol was as follows: thawing dishes were prepared 24 h in advance by adding 1 mL blastocyst culture medium, overlaying with sterile mineral oil, and equilibrating in the incubator for > 6 h. On the day of thawing, the thawing solution (TS) was pre-warmed at 37 °C for 60 min, while dilution solution (DS), washing solution 1 (WS1), and washing solution 2 (WS2) were equilibrated at room temperature for ≥ 45 min. For thawing, 500 µL of pre-warmed TS was transferred to a 60 mm culture dish. Using precooled forceps, the vitrification carrier was rapidly retrieved from liquid nitrogen and immersed in TS, with gentle agitation to release embryos within 60 s. The embryos were then sequentially transferred to DS for 3 min, a 100 µL droplet of WS1 for 5 min, and a 100 µL droplet of WS2 for an additional 5 min, followed by a final wash in pre-equilibrated culture medium before transfer to pre-equilibrated microdrops using a Pasteur pipette. For assisted hatching, zona pellucida perforations were created using a non-contact laser, and embryos were incubated until ICM isolation.

### ICM isolation

Microdrops (each containing 30 µL fertilization medium and covered with mineral oil) were prepared one day in advance and equilibrated in the incubator for > 6 h. The embryos were transferred into the microdrops 1 h prior to ICM isolation. The holding pipette and biopsy needle were rinsed with 30 µL of PVP solution, then washed with 30 µL of fertilization medium to remove excess PVP. Using the holding pipette, trophoblast cells distal to the ICM were positioned, and the embryo was rotated to visualize the ICM boundary. The biopsy needle was used to aspirate trophoblast cells adjacent to the ICM. A 30–40 ms laser pulse was applied to perforate trophoblast cells near the ICM, followed by gentle separation of the ICM from the trophoblast cells. The isolated ICM was then used for hESC derivation.

### hESC derivation

The isolated ICMs were plated into laminin-521 (0.50 µg/cm²)-coated dishes in TeSR™-AOF medium containing 10 µM Y27632 and incubated at 37 °C with 5% CO₂. For ICMs invisible blastocysts, all biopsy-derived cellular materials were collected and plated for hESC derivation. Culture medium was not refreshed during the first 4 days post-plating (dpp) to facilitate ICM adhesion. Starting at 5 dpp, medium (TeSR™-AOF with 10 µM Y27632) was changed every 2 days. From 7 dpp onward, Y27632 was removed, and medium was refreshed daily. Primary hESC clones could be passaged mechanically after 12 dpp. After Passage 1, hESCs could be passaged using the commercially GMP-grade enzymatic solution ReleSR according to the instructions. hESC lines at Passage 3 could be harvested and cryopreserved in a batch of 10 vials as seed stocks with about 5 × 10^5^ to 2 × 10^6^ viable cells per vial in the GMP-grade cryopreservation medium CryoStar CS10. Clinical-grade hESC lines were derived in an A-grade environment in a B-grade room, and the GMP-grade regents used for hESC derivation are listed in Additional file 2 Table S1.

### Colony formation assay

hESCs in the logarithmic growth phase were dissociated into single cells using Accutase and resuspended in either TeSR™-AOF medium or E8 medium, both supplemented with 10 µM Y27632. Cells were adjusted to 300 cells/well and seeded into 12-well plates pre-coated with either laminin-521 (0.75, 0.50, and 0.25 µg/cm²) or vitronectin (3, 2, and 1 µg/cm²). After 7 days of culture, colonies were fixed with 4% paraformaldehyde and stained with 0.1% crystal violet. Images were acquired, and the colony area was quantified using Image J software.

### Alkaline phosphatase staining

Alkaline phosphatase staining was performed using the Alkaline phosphatase color development kit as described by the manufacturer. The cells were washed with DPBS and stained with alkaline phosphatase for 20 min, and washed three times with pure water and photographed.

### Flow cytometry

Single cells were fixed with 4% paraformaldehyde for 15 min, treated with permeabilization buffer for 15 min and blocked with a blocking buffer for 30 min. Cell samples were incubated with primary antibody at 4 ℃ for 1 h and fluorophore-conjugated secondary antibody at room temperature for 30 min. 10,000 events per sample were acquired using a flow cytometer with gating based on the negative control and the different gene expression patterns of each cell population. The antibodies used are listed in Additional file 2 Table S2.

### Teratoma formation

All animal experiments were approved by the Animal Ethics Committee of Hangzhou Institute of Medicine, Chinese Academy of Sciences and conducted in accordance with the guidelines (Ethical No. AP2024-10-0307). SCID-Beige mice (6–8 weeks old, male) were purchased from Charles River Laboratories and housed at a constant room temperature of 23 ± 2 ℃ with a 12:12 light/dark cycle and free access to water and food in a specific pathogen-free (SPF) condition. For teratoma formation, approximately 2 × 10^6^ hESCs were harvested by Accutase, and resuspended in a 1:1 mixture of cold Matrigel and culture medium. Then the cells were subcutaneously injected to SCID-Beige mice which were anesthetized with isoflurane. When the teratomas reached 10 mm in diameter, the animals were euthanized via CO_2_ exposure. Subsequently, the teratomas were harvested and sectioned for hematoxylin and eosin (H&E) staining. The removed teratoma was put into the tissue fixative and embedded by Wuhan Servicebio Technology Co., Ltd. for sectioning and H&E staining.

### Doubling time

Cell proliferation was analyzed using a Cell Counting Kit-8 (CCK8). hESCs were plated into matrix-coated 96-well plates at a density of 5,000 cells per well and cultured in incubator at 37 °C, 5% CO_2_. On days 2, 3, and 4, 10% (v/v) CCK8 reagent was added to each well. After 2 h of incubation, absorbance was measured at 450 nm using a microplate reader. Five replicate wells were analyzed per time point, while two replicate wells were set up as blank controls at each time point. The number of cells was calculated according to the formula. Cell doubling time: Td = t × Log (2) / Log (N_t_ / N_0_). t: incubation time; N_0_: initial cell number; N_t_: the number of cells at t.

### STR analyses

Short tandem repeat (STR) was tested by Shanghai Biyuntian Biotechnology. Briefly, genomic DNA were extracted from the cells and STR loci were amplified via PCR to generate specific fragments. The amplified products were separated using capillary electrophoresis, and the number of repeat units at each STR locus was detected to generate a unique STR profile. The STR profile were analyzed and compared with standard databases (ATCC) to confirm the genetic identity, purity, and the absence of interspecies contamination.

### Karyotyping

For karyotype analysis, well-dispersed mid-stage chromosomes were obtained by cell culture and mid-stage blocking. The chromosomes were stained to develop bands, and high-definition images are taken through a microscope. Arrange the karyotype diagrammed by size and banding type, and compared with the standard pattern to recognize numerical abnormalities or structural aberrations.

### Pancreatic β cells differentiation

The differentiation of hESCs into β cells was performed using a six-stage protocol, as previously described [16]. Briefly, hESCs were dissociated into single cells using Accutase and resuspended in mTESR1 medium supplemented with 10 µM Y27632. The cells were then plated at a density of 1.5 × 10⁵ cells/cm² on Matrigel-coated surfaces. Differentiation was initiated 24 h post-seeding with specific media formulations for each stage.

*Stage 1 (4 days)* On day 1, cells were maintained in MCDB 131 supplemented with 1 × Glutamax, 4.5 mM glucose, 1% B27, 0.25 mM ascorbic acid, 100 ng/mL Activin A, and 6 µM CHIR99021. From days 2 to 4, the medium was replaced with MCDB 131 containing 1 × Glutamax, 4.5 mM glucose, 1% B27, and 100 ng/mL Activin A.

*Stage 2 (2 days)* Cells were treated with MCDB 131 supplemented with 1 × Glutamax, 4.5 mM glucose, 1% B27, 0.25 mM ascorbic acid, 50 ng/mL KGF, 100 nM Wnt-C59, and 5 µM SB431542.

*Stage 3 (4 days)* Cells were cultured in DMEM containing 1% B27, 0.25 mM ascorbic acid, 0.25 mM SANT-1, 2 µM retinoic acid, and 100 nM LDN193189. At the end of Stage 3, cells were dissociated using Accutase for 5–8 min at 37 °C, rinsed with DMEM, and plated at 6 × 10⁶ cells/well in six-well AggreWell Microwell Plates in Stage 4 medium supplemented with 10 µM Y27632. After 24 h, the clusters were transferred to ultra-low-attachment six-well plates and cultured in an incubator shaker at 90 rpm, 37 °C, 5% CO₂, and 85% humidity.

*Stage 4 (6 days)* Cell clusters were maintained in DMEM containing 1% B27, 0.25 mM ascorbic acid, 100 ng/mL EGF, 200 nM TPB, 0.25 mM SANT-1, and 10 mM Nicotinamide.

*Stage 5 (6 days)* Clusters were further cultured in DMEM enriched with 1% B27, 0.25 mM ascorbic acid, 10 µM ALK5 inhibitor II, 300 nM LDN193189, 1 µM T3, 10 µM ISX9, 10 µg/ml heparin, 100 nM γ-secretase inhibitor XXI, 100 nM Wnt-C59, and 10 µM Y27632. ISX9 was included from days 1 to 3.

*Stage 6 (2 days)* Cells from Stage 5 were treated with DMEM supplemented with 1% B27, 0.25 mM ascorbic acid, 10 µM ALK5 inhibitor II, 0.5 µM R428, 1 µM T3, 10 µg/mL heparin, 10 µM zinc sulfate, and 2 mM N-Acetyl-L-cysteine.

The regents used are listed in Additional file 2 Table S3.

### Glucose-stimulated insulin secretion

hESC-islets (10–20 clusters) were collected into a 12-well plate and rinsed twice with Krebs buffer (129 mM NaCl, 4.8 mM KCl, 2.5 mM CaCl₂, 1.2 mM MgSO₄, 1 mM Na₂HPO₄, 1.2 mM KH₂PO₄, 5 mM NaHCO₃, 10 mM HEPES, and 0.1% BSA in deionized sterile-filtered water). The clusters were then incubated sequentially in Krebs buffer containing 2.8 mM glucose and Krebs buffer with 16.7 mM glucose for 1 h each. After each incubation, the supernatant was collected, and the clusters were rinsed with fresh Krebs buffer during solution changes. Supernatant samples were frozen at -80 °C until analysis. Following the assay, cells were dissociated into single cells using Accutase and counted with the Countess II Automated Cell Counter.

### Cardiomyocyte differentiation

The hESC-derived cardiomyocytes were generated using a 2D monolayer differentiation protocol [17]. Briefly, Undifferentiated hESCs were dissociated and replated into a Matrigel-coated 6-well plate at a density of 1.5 × 10^4^ cells/cm². The hESCs were cultured and expanded to 85% confluence, and then treated with 6 µM CHIR99021 in RPMI 1640 with 1% B27 supplement minus insulin (RPMI + B27-Insulin) for 2 days. On day 3, cells were placed in RPMI + B27-Insulin without CHIR99021. On days 4–5, cells were treated with 5 µM IWR-1 to inhibit Wnt signaling pathway. On days 5–6, cells were removed from IWR-1 treatment and placed in RPMI + B27-Insulin. From day 7 onwards, cells were placed and cultured in RPMI 1640 (without glucose) and B27 supplement with insulin until beating cells were observed.

The regents used are listed in Additional file 2 Table S3.

### Corneal endothelial cell differentiation

The corneal endothelial cell differentiation protocol was slightly modified from that of a previously described [18]. Briefly, hESCs were plated at a density of 2.5 × 10⁵ cells per well on Matrigel-coated 12-well plates and cultured until reaching 30%, then cultured in neural crest differentiation medium consisting of DMEM/F12, 20% knockout serum replacement, 4 ng/mL bFGF, and 1 µM retinoic acid. After 5 days of culture, the medium was replaced with corneal endothelial differentiation medium consisting of DMEM/F12, 8 ng/mL bFGF, 10 ng/mL PDGF-BB, 10 ng/mL DKK-2, 50 µg/mL ascorbic acid, 10 ng/mL Heregulin β-1, 200 ng/mL IGF-1, 1 × B27, 0.01 mM β-mercaptoethanol, 10 µM Y27632, and 1 µM SB431542. To induce corneal endothelial cells, the neural crest cells were cultured for 10 days. The cells were cultured at 37 °C and 5% CO_2_ during differentiation and the medium was changed every day.

The regents used are listed in Additional file 2 Table S3.

### RNA extraction and quantitative PCR

Cell lysis was performed by adding 1 mL of RNAiso Plus to each sample, followed by vigorous vortex mixing for 30 s to ensure complete lysis. Samples were then incubated on ice for 10 min. For phase separation, 200 µL of chloroform was added to lysate. Tubes were vigorously shaken for 30 s and subsequently incubated on ice for 5 min to facilitate complete phase separation. The mixture was centrifuged at 12,000 g for 10 min at 4 °C, and the aqueous phase was carefully transferred to a fresh tube. RNA was precipitated by mixing with 500 µL of isopropanol and incubating on ice for 10 min, followed by centrifugation at 12,000 g for 10 min at 4 °C. The resulting RNA pellet was washed with 1 mL of ice-cold 75% ethanol, centrifuged at 7,500 g for 5 min at 4 °C, and air-dried before resuspension in DEPC-treated water. RNA concentration and purity were measured using a Nanodrop spectrophotometer. Reverse transcription was performed using the PrimeScript RT reagent Kit with gDNA Eraser, followed by qPCR using TB Green^®^ Premix Ex Taq according to the manufacturer’s instructions. The primers are listed in Additional file 2 Table S4.

### Immunofluorescence staining

Cell cultures or tissue sections were washed with PBS and fixed with 4% paraformaldehyde for 15 min at room temperature. The sample was washed twice with PBS (5 min each), permeabilized with 0.2% Triton X-100 in PBS at room temperature for 30 min, and then blocked with 5% BSA in PBS at room temperature for 1 h. Primary antibody was incubated for overnight incubation at 4 ℃ and secondary antibody (containing Hoechst33342) was incubated at 37 ℃ for 1 h. The sample was washed 3 times for 5 min each, and PBS was added for microscopic observation and photographic recording. The antibodies used are listed in Additional file 2 Table S2.

### Sterility

The sterility was referred to the Pharmacopoeia of the People’s Republic of China and was detected by cultural method. The sterility tests were performed with BACT/ALERT^®^ (BioMérieux). After 24 h of incubation with cells, the conditioned medium was collected, placed in culture bottles for further incubation. Both aerobic and anaerobic testing were performed. After 14 days, the presence or absence of any contamination was confirmed.

### Mycoplasma

Mycoplasma was detected by PCR. Before the passaging, the supernatant of cell culture was collected, which was heated at 95 ℃ for 10 min and then centrifuged at 12,000 g for 1 min. The supernatant was used as a template for PCR amplification using specific primers. The amplified products were photographed after gel electrophoresis.

### Virus

Virus detection by fluorescent quantitative PCR with TaqMan probe. Primers and probes were designed to target the conserved sites. The viability of primers and probes was tested by gradient dilution of positive fragments to obtain the correspondence equations. The cDNA and DNA of cell lines were then used as templates to detect the viral contamination.

### Endotoxin

Endotoxin was tested by Hangzhou Chain Medical Laboratory according to the colorimetric method. The chromogenic substrate in the coagulase hydrolysis reaction, which causes a change in absorbance, is used to quantify the concentration of gram-negative bacterial lipopolysaccharides based on the dynamic detection of the rate of change in the absorbance of the solution.

### Statistical analyses

T-test and One-way ANOVA were performed using Graphpad Prism 10. P values are presented as follows: ^*^, *P* < 0.05; ^**^, *P* < 0.005; ^***^, *P* < 0.0005; ns, not significant.

The work has been reported in line with the ARRIVE guidelines 2.0.

## Results

### Establishment of a protocol for the derivation of hESCs

**Fig. 1:**
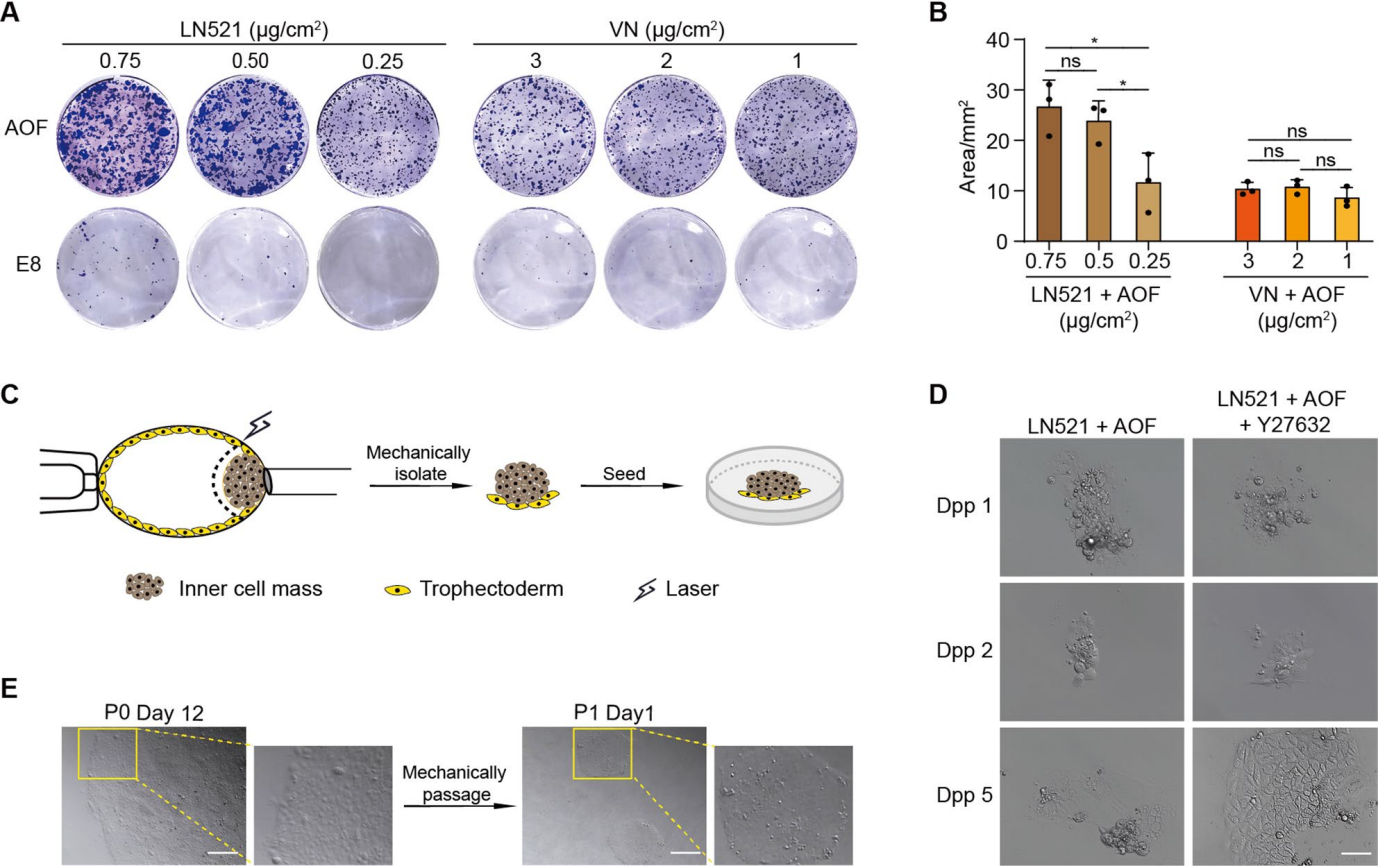
Protocol optimization for hESC establishment. **A**-**B** Crystalline violet staining (**A**) and quantitative analysis (**B**) of hESC clones in AOF and E8 media with different concentrations of LN521 or VN, respectively. *n* = 3. The results were presented as mean ± SD. ^*^, *p* < 0.05; n.s, not significant. **C** Diagram of laser-assisted isolation of ICM from blastocyst. The dotted line represents the laser cutting trajectory. **D** Primary clone formation on LN521 in AOF with or without Y27632 treatment. Dpp, days post-ICM plating. Scale bar, 50 μm. **E** 12 days post-ICM plating, primary hESC clone formed and could be passaged mechanically. Morphology of primary hESC clone (P0) and hESC clones at Passage 1 (P1). The small image on the right shows an enlargement of the yellow rectangle in the left large image. Scale bar, 100 μm

To develop a clinically compliant protocol for hESC derivation under feeder- and xeno-free conditions, we first evaluated the extracellular matrix (ECM) components and hPSC culture media, the two critical parameters that affect cell survival and clonogenic efficiency of hPSCs. The tested ECM components and hESC culture media were selected based on clinical-grade specifications, including chemically defined composition, xeno-free origin, GMP compliance, and commercial availability. The ECM components laminin-521 (LN521) and vitronectin (VN), which fully satisfy every requirement outlined above, were selected as the candidates for testing. The established hESC lines at early passages were utilized for testing colony forming ability. Our results showed that LN521 exhibited superior support for cell adhesion and growth in a concentration-dependent manner. Specifically, increasing the LN521 coating density from 0.25 to 0.50 µg/cm² achieved a 2-fold increase in cell number (Fig. 1A and B). Evaluation of the culture media revealed that albumin-supplemented TeSR-AOF (AOF) medium significantly enhanced colony formation and cell growth while maintaining the typical morphology of hESCs across both LN521- and VN-coated plates (Fig. 1A and B). Accordingly, a LN521-coating density of 0.5 µg/cm² in combination with albumin-supplemented AOF medium was selected for subsequent hESC derivation.

The study of hESC derivation was approved by the Medical Ethics Committee of Affiliated Hospital of Yunnan University (Ethical No. 2023224). All embryos used in the study were frozen and clinically discarded, which were surplus to requirement or unsuitable for clinical use due to poor development. A fully traceable process of informed ethical consent was obtained from all couples who donated their embryos in accordance with ethical guidelines. Donor candidates underwent multiphasic medical evaluations including infectious disease panels (HIV-1/2, HBV, HCV and TP), karyotype and detailed family histories to identify potential oncological, hereditary, and congenital disorders. Eligible embryos were recruited for hESC derivation.

To avoid potential xeno-contamination risks inherent in immunosurgical protocols, we developed a laser-assisted ICM isolation method for precise ablation of trophectoderm (Fig. 1C). This mechanical dissection method was optimized through adaptation of standardized preimplantation genetic diagnosis (PGD) biopsy workflows, utilizing micromanipulation systems with biopsy needles. Notably, all isolation procedures exclusively employed clinical-grade reagents and consumables, including in vitro fertilization (IVF)-validated culture media and embryo-tested mineral oil, thereby ensuring compliance with GMP standards for therapeutic-grade hESC derivation. For morphologically distinct ICMs (grade A and B), they were selectively microsurgically excised with removal of most trophectoderm cell mass. For developmentally compromised blastocysts with invisible ICMs (grade C), all biopsy-derived cellular materials were collected and plated for hESC line derivation.

After mechanical isolation from blastocysts, ICMs were plated on LN521 in AOF medium with ROCK inhibitor Y27632. GMP-grade Y27632 was utilized for clinical-grade hESC lines derivation. Morphological monitoring showed that primary ICM clusters effectively attached to LN521 within 24 h (Fig. 1D). However, gradual cell death was observed after Y27632 removal from day 2 onward. Therefore, Y27632 treatment was extended throughout the first week. The results indicated that sustained Y27632 treatment significantly enhanced the survival and proliferation of primary pluripotent stem cells derived from ICMs (Fig. 1D). After 5–7 days in culture, small clusters of cells exhibiting typical hESC morphology emerged (Fig. 1D). Subsequent expansion of hESC clones no longer required Y27632. These findings suggest that ROCK inhibition is essential for the initial adhesion, survival, and expansion of primary hESCs under feeder-free conditions. The primary hESC clones could be mechanically dissociated at 12–16 days post-plating (Fig. 1E). From Passage 2, hESCs could be maintained on LN521 in AOF medium and propagated as small clumps using a GMP-grade xeno-free enzymatic solution. Over 20 million hESCs could be obtained at Passage 3 and hESC seed stocks could be cryopreserved at this stage or subsequently.

**Fig. 2:**
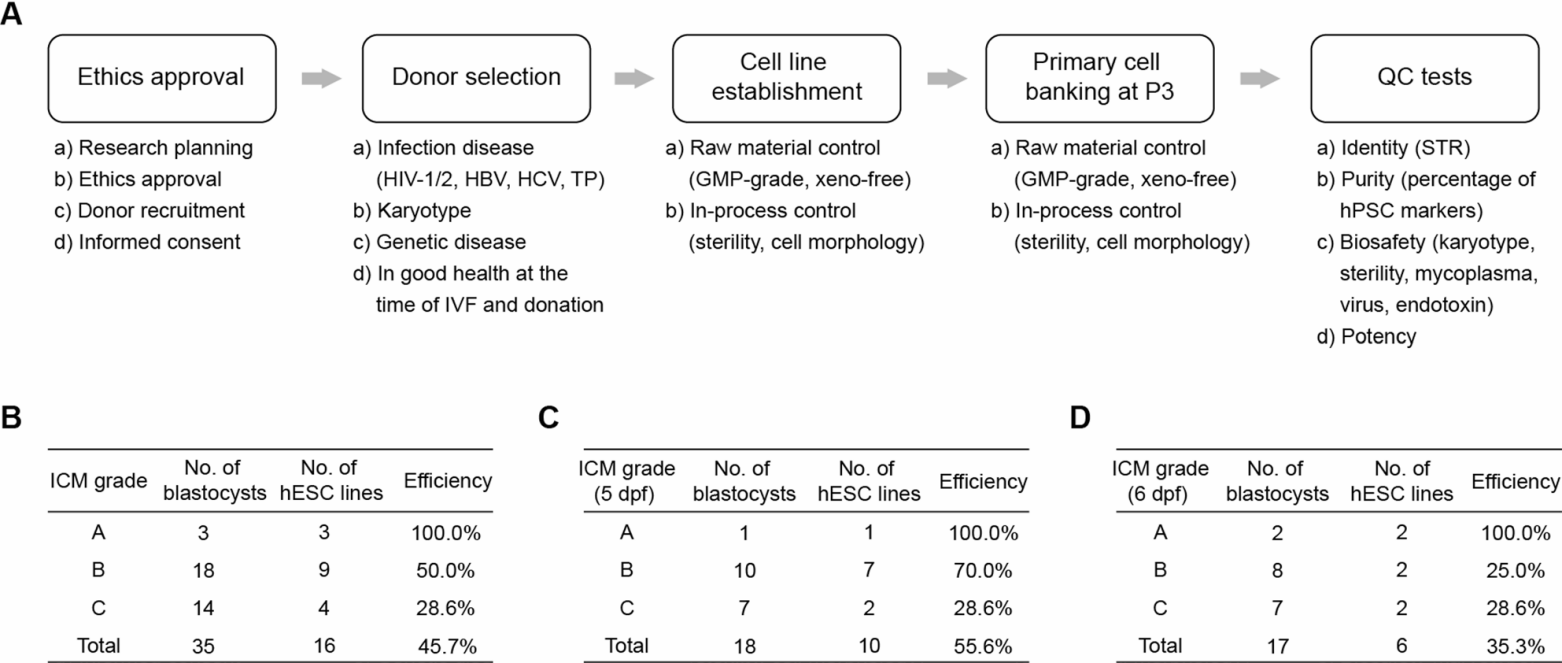
Workflow of establishment of clinical-grade hESCs. **A** Process for the derivation and characterization of clinical-grade hESC lines. **B**-**D** Derivation efficiencies of hESC lines. A total of 35 blastocysts were used in this study (**B**), including 18 blastocysts at 5 dpf (**C**) and 17 blastocysts at 6 dpf (D). QC, quality control. Dpf, days post-fertilization

After optimizing the culture condition and ICM isolation protocol, we established a standardized pipeline for hESC derivation (Fig. 2A). All reagents used were of GMP-grade and commercially available (Table S1). Using this protocol, we successfully derived 16 hESC lines from 35 eligible blastocysts, achieving an overall efficiency of approximately 45.7% (Fig. 2B). Our results showed a positive correlation between derivation efficiency and ICM quality (Fig. 2B-D). Notably, 5-day post-fertilization (dpf) blastocysts showed a higher derivation rate compared to 6-dpf embryos (Fig. 2C-D). For 5-dpf blastocysts with visible ICMs (grades A and B), the derivation efficiency exceeded 70% (Fig. 2C). Even for blastocysts with barely visible ICMs (grade C), we achieved a derivation efficiency of approximately 30% (Fig. 2B). Collectively, these findings suggest that our feeder- and xeno-free protocol effectively supports efficient hESC derivation from blastocysts.

### Establishment and characterization of clinical-grade hESCs

Following the workflow presented in Fig. 2A, we derived two clinical-grade hESC lines (HES1 and HES2). The process included informed ethical consent, donor eligibility determination, details derivation procedures, in-process testing and quality control of the derived hESCs. The clinical-grade hESCs were generated in an A-grade environment in a B-grade facility. All reagents used for hESC derivation, propagation and cryopreservation were xeno-free and of GMP-grade, with verification through specific tests detailed in the certificate of analysis (COA) (Table S1). Standard operating procedure (SOP) and record forms for derivation were executed in accordance with GMP requirements.

**Fig. 3:**
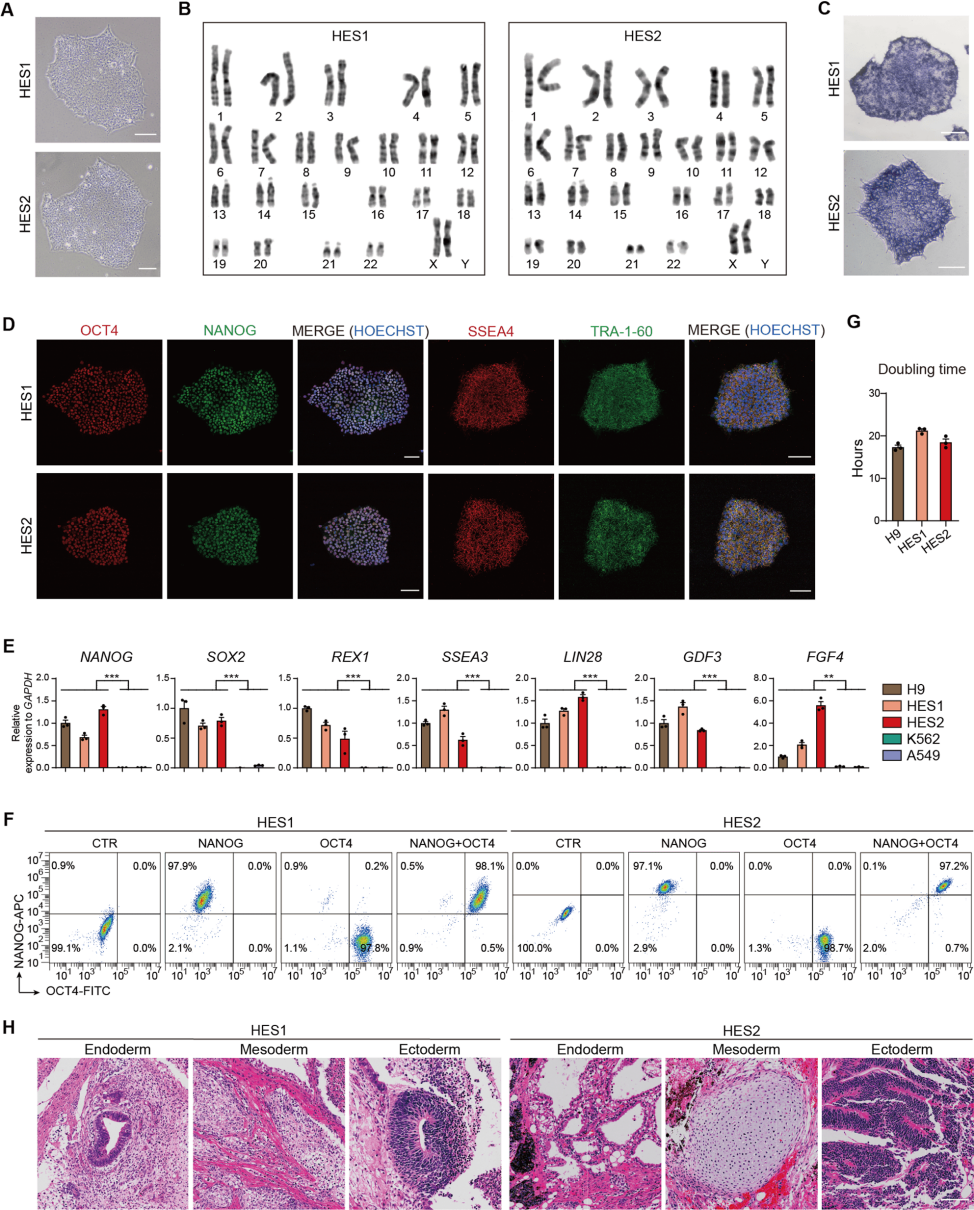
Characterization of HES1 and HES2. **A**-**C** Morphology (**A**), karyotype analysis (**B**), and alkaline phosphatase staining (**C**) of HES1 and HES2. Scale bars, 100 μm. **D** The expression of pluripotency markers was analyzed via immunofluorescence. Transcription factors OCT4 (red) and NANOG (green) and cell surface markers SSEA4 (red) and TRA-1-60 (green) were detected. Nuclei were counterstained with Hoechst33342 (blue). Scale bars, 100 μm. **E** The expression levels of pluripotency genes (*NANOG*,* SOX2*,* REX1*,* SSEA3*,* LIN28*,* GDF3*, and *FGF4*) were assessed via qRT-PCR. The human embryonic stem cell line H9 (positive control) and the differentiated cell lines K562 (a human leukemia cell line) and A549 (a human lung adenocarcinoma cell line) were included to validate assay specificity. *n* = 3. ^**^, *p* < 0.005; ^***^, *p* < 0.0005. **F** The purity was assessed by flow cytometry. **G** Population doubling time analysis for HES1 and HES2. *n* = 3. **H** Teratoma formation. All three germ layer tissues were detected in H&E-stained teratoma sections. Scale bars, 50 μm. The results were presented as mean ± SD

HES1 and HES2 were fully characterized. HES1 and HES2 showed the typical morphology of human pluripotent stem cells (Fig. 3A). Biosafety tests performed at Passage 5 including sterility, mycoplasma, virus (HIV-1/2, HBV, HCV, TP, HTLV-1, HCMV, EBV, HHV6, and HHV7), and endotoxin testing, confirmed the safety profile of these cell lines (Table 1). Short tandem repeat (STR) profiling confirmed the absence of cellular cross-contamination between hESC lines (Figure S1). Pluripotency characterization was conducted around Passage 10. HES1 and HES2 showed normal karyotype, were alkaline phosphatase positive and expressed typical hPSC markers (Fig. 3B-E). Flow cytometry analysis of OCT4 and NANOG revealed a high purity of HES1 and HES2 (> 97%) (Fig. 3F). The proliferation rates of HES1 and HES2 was comparable to H9 (Fig. 3G). When transplanted into the immunodeficient mice, these cells formed teratomas, which contained tissues from all three germ layers (Fig. 3H). Collectively, these results showed that HES1 and HES2 represent high-quality hESC seeds suitable for establishing clinical-grade master cell banks for future therapeutic applications.

**Table 1:** Biological safety tests

Sterility and pathogen	HES1	HES2
Bacteria and fungi	-	-
Mycoplasma	-	-
Human immunodeficiency virus I (HIV-1)	-	-
Human immunodeficiency virus II (HIV-2)	-	-
Epstein-barr virus (EBV)	-	-
Human hepatitis C virus (HCV)	-	-
Human cytomegalovirus (HCMV)	-	-
Human t-lymphotropic virus I (HTLV-1)	-	-
Human hepatitis B virus (HBV)	-	-
Human herpesvirus 6 (HHV6)	-	-
Human herpesvirus 7 (HHV7)	-	-
Treponema pallidum (TP)	-	-
Endotoxin level	< 0.01 EU/mL	< 0.01 EU/mL

### Direct differentiation of clinical-grade hESC

**Fig. 4:**
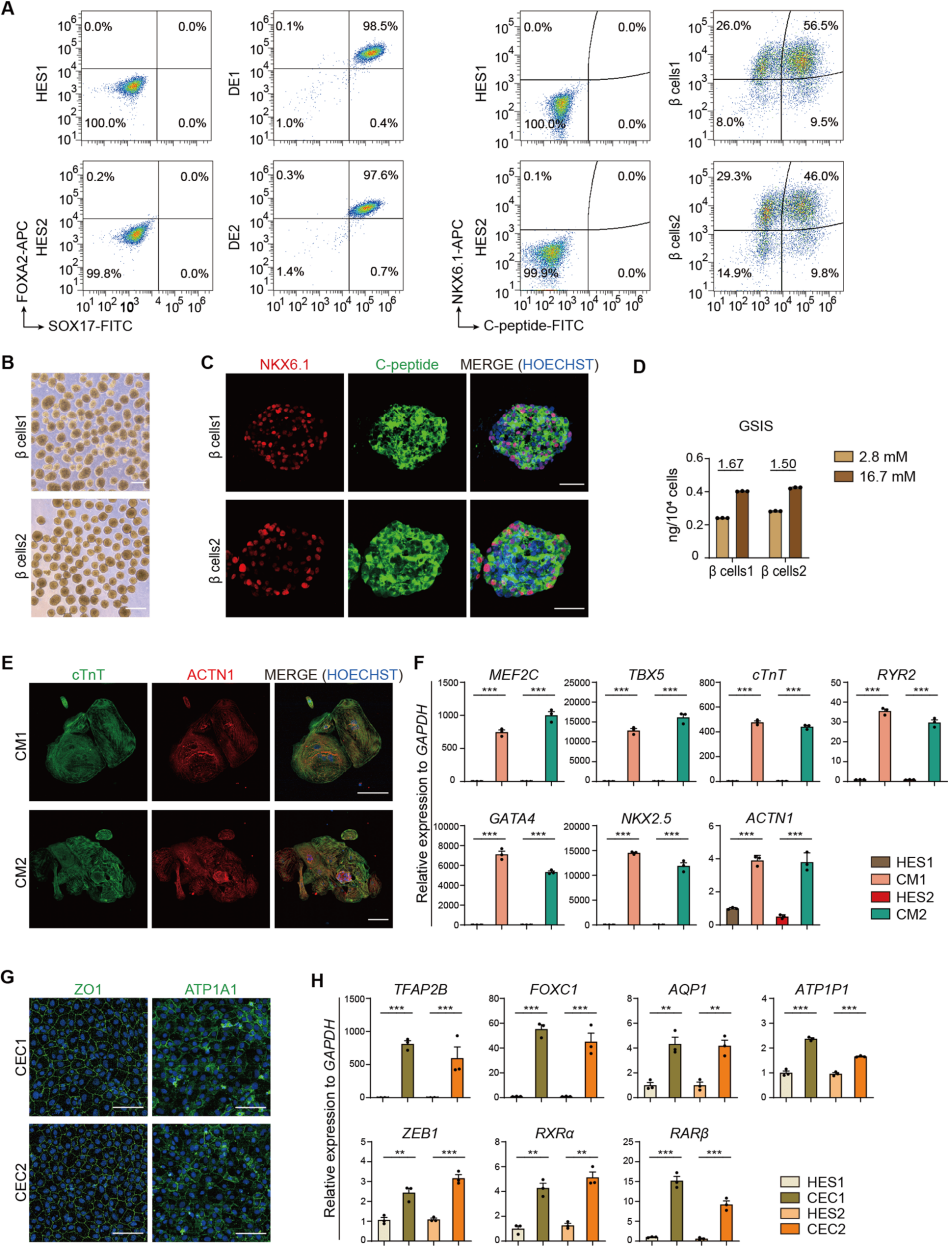
HES1 and HES2 are capable of differentiation into three germ layer lineages. **A** Flow cytometry detection of endodermal progenitor cell markers (FOXA2 and SOX17) and pancreatic β cell markers (NKX6.1 and C-peptide) at Stage 1 and Stage 6 during pancreatic β cell differentiation of HES1 and HES2. DE1, HES1-derived definitive endodermal cells (Stage 1). DE2, HES2-derived definitive endodermal cells (Stage 1). β cells1, HES1-derived pancreatic β cells (Stage 6). β cells2, HES2-derived pancreatic β cells (Stage 6). **B** Morphology of differentiated pancreatic islet at Stage 6. Scale bars, 300 μm. **C** Immunofluorescence staining of β cell markers. Nuclei were stained with Hoechst33342 (blue). Scale bars, 50 μm. **D** Glucose stimulated insulin secretion (GSIS) assay. The insulin secretion level of HES1- and HES2-derived β cells were measured at low (2.8 mM) and high (16.7 mM) glucose concentrations. *n* = 3. The glucose-stimulated index values are indicated above the bar. **E** Immunofluorescence staining of cardiomyocyte markers (cTNT and ACTN) of HES1- and HES2-derived cardiomyocytes. Nuclei were stained with Hoechst33342 (blue). CM1, HES1-derived cardiomyocytes. CM2, HES2-derived cardiomyocytes. Scale bars, 100 μm. **F** The expression of cardiomyocyte marker genes was detected by qRT-PCR. *n* = 3. ^**^, *p* < 0.005; ^***^, *p* < 0.0005. **G** Immunofluorescence staining of corneal endothelial cell markers (ZO1 and ATP1A1). Nuclei were stained with Hoechst33342 (blue). CEC1, HES1-derived corneal endothelial cells. CEC2, HES2-derived corneal endothelial cells. Scale bars, 100 μm. **H** The expression of corneal endothelial cell marker genes was detected by qRT-PCR. *n* = 3. The results were presented as mean ± SD. ^**^, *p* < 0.005; ^***^, *p* < 0.0005

Next, we directed HES1 and HES2 differentiation toward specific cell types across all three germ layers. We first differentiated HES1 and HES2 into pancreatic β cells, a representative endodermal lineage, using the previously established protocol [16]. Stage 1 differentiation yielded endodermal progenitors with >90% co-expression of FOXA2 and SOX17 (Fig. 4A). By Stage 6, cell clusters exhibiting typical islet morphology were observed (Fig. 4B). Approximately 50% of the differentiated cells were pancreatic β cells, comparable to that of primary human islets (Fig. 4A) [19]. Immunofluorescence staining confirmed the expression of typical β cell markers (Fig. 4C). Furthermore, the insulin secretion of differentiated β cells was responded to glucose stimulation (Fig. 4D).

For mesoderm differentiation, we generated cardiomyocytes from HES1 and HES2 using an established protocol [17]. Immunofluorescence staining showed the expression of functional marker genes ACTN1 and cTNT (Fig. 4E). Master transcriptional regulators of cardiac lineage and typical functional genes, such as *MEF2C*,* TBX5*,* GATA4*,* NKX2.5*,* cTNT*,* RYR2*, and *ACTN1* were significantly up-regulated after the formation of beating cell clusters (Fig. 4F).

For ectoderm differentiation, neural crest-derived corneal endothelial cells were generated from HES1 and HES2 using an established protocol from HES1 and HES2 [18]. Initial neural crest commitment (Stage 1) was followed by corneal endothelial specification (Stage 2), yielding uniform hexagonal cells expressing tight junction protein ZO1 and the sodium-potassium cotransporter ATP1A1 after 3 weeks induction (Fig. 4G). qRT-PCR analysis confirmed upregulation of corneal endothelial markers (Fig. 4H).

Collectively, these results demonstrate robust trilineage differentiation capacity of both HES1 and HES2, enabling the generation of functional cells across all three germ layers, endodermal (pancreatic β cells), mesodermal (cardiomyocytes), and ectodermal (corneal endothelial cells), using published differentiation protocols.

## Discussion

In this study, we established a standardized protocol that enabled the effective derivation of hESC lines under defined, feeder- and xeno-free conditions. This derivation protocol is operationally simple and easily standardized, with the reagents commercially available and of GMP-grade, meeting the critical requirements for clinical-grade cell production. The high efficiency and ease of standardization make it suitable for adoption in both clinical application and basic research.

Using clinically surplus and discarded frozen-thawed embryos, we successfully established 16 hESC lines, achieving an average derivation efficiency of 45.7%. Notably, for blastocysts with discernible ICMs of grades A and B, the derivation efficiency reached approximately 60%, with all three grade A ICMs successfully yielding hESC lines. Remarkably, our protocol also enabled the derivation of hESC lines from embryos with poorly developed ICMs (grade C), which are typically deemed unsuitable for clinical use, with a derivation efficiency nearing 30%. This aligns with previous studies that observed a positive correlation between ICM quality and derivation efficiency [9]. When compared to recent advances in hESC derivation reporting success rates of 7.9% to 45.5% under feeder- and xeno-free conditions, our protocol showed superior efficiency (Table S5) [14, 15]. Notably, we found that using 5-dpf blastocysts resulted in higher derivation efficiency and the derivation efficiency decreased with prolonged culture periods, which phenomenon was also observed in previous feeder-layer-based studies [9]. In the IVF context, extended culture periods are often linked to decreased embryo developmental rate and overall quality, which could impact the capacity to derive stable hESC lines [20, 21]. Collectively, the ability to efficiently establish hESCs from low-quality, clinically discarded embryos enable us to derive normal and disease-specific cell lines for basic research and clinical applications.

The higher derivation efficiency could be due to the optimized ICM isolation method and cell culture conditions. The traditional ICM isolation methods include enzymatic isolation and conventional mechanical isolation. For the enzymatic isolation, the reagents are predominantly animal-derived, creating a major barrier to the production of clinical-grade hESCs [22]. The proteolytic enzymes most often used are prepared from animal tissues. Furthermore, the immunosurgery protocol depends on rabbit anti-human antiserum and guinea-pig complement serum to lyse trophectoderm cells. In comparison, all the operation procedures related to laser-assisted ICM isolation was accomplished in blastocyst culture medium, which is clinical-grade and xeno-free. Compared with the conventional mechanical isolation method, laser-assisted ICM isolation allowed more precise operation and more complete removal of trophoblasts. Previous studies have shown that minimized proliferation of trophoblast cells facilitated the derivation of hESC lines on feeder layers [13]. The suppression of trophoblast cells allowed for the expansion of primary pluripotent stem cells in ICMs during the first few days. Furthermore, our results showed that Y27632 treatment in the first week of derivation was also crucial for the survival and expansion of primary hESC clones. Unlike ICMs attached to feeder layers, which initiated proliferation and formed dome-like structures, ICMs plated on LN521-coated dishes fully adhered and flattened of domed ICMs [23]. The adaption of primary ICM cells on LN521-coated dishes could involve cytoskeletal reorganization, during which Y27632 was indispensable for cell survival and initial expansion. Once primary clone formation and stable expansion were achieved, Y27632 was no longer required for routine cell culture. Previous studies have demonstrated that Y27632 facilitated the survival of dissociated hESCs by regulating cytoskeleton reorganization, suggesting its relevance for maintaining the viability of primary pluripotent stem cell within ICMs without feeder layer [24].

## Conclusion

In summary, we demonstrate an advanced method for effective and standardized establishment of hESC lines under feeder- and xeno-free conditions. As the pluripotent stem cells naturally occurring during the embryonic development, hESCs remain an irreplaceable cell source for both basic research and clinical applications. Efficient derivation, propagation and banking of hESC lines from frozen-thawed normal and disease embryos would offer a valuable cell source for advancing regenerative medicine, disease modeling, and therapeutic development.

## Supplementary Information

Below is the link to the electronic supplementary material.

Supplementary Material 1.

Supplementary Material 2.

## Data Availability

The data that support the findings of this study are available from the corresponding author upon reasonable request and within its Supplementary Data published online. All additional files are included in the manuscript.

## Abbreviations

hESCs: Human embryonic stem cells
ICM: Inner cell mass
GMP: Good Manufacturing Practice
hPSCs: Human pluripotent stem cells
STR: Short tandem repeat
ECM: Extracellular matrix
LN521: Laminin-521
VN: Vitronectin
PGD: Preimplantation genetic diagnosis
IVF: In vitro fertilization
Dpf: Days post-fertilization
COA: Certificate of analysis
SOP: Standard operating procedure
Dpp: days post ICM plating
QC: Quality control
