# Supplementary figures and images for: A simple and standardized method supports efficient derivation of clinical-grade human embryonic stem cells under feeder- and xeno-free conditions
Source: Stem Cell Res Ther. 2025 Dec 1;17:9. doi: 10.1186/s13287-025-04831-3 (PMC12772070; doi:10.1186/s13287-025-04831-3)

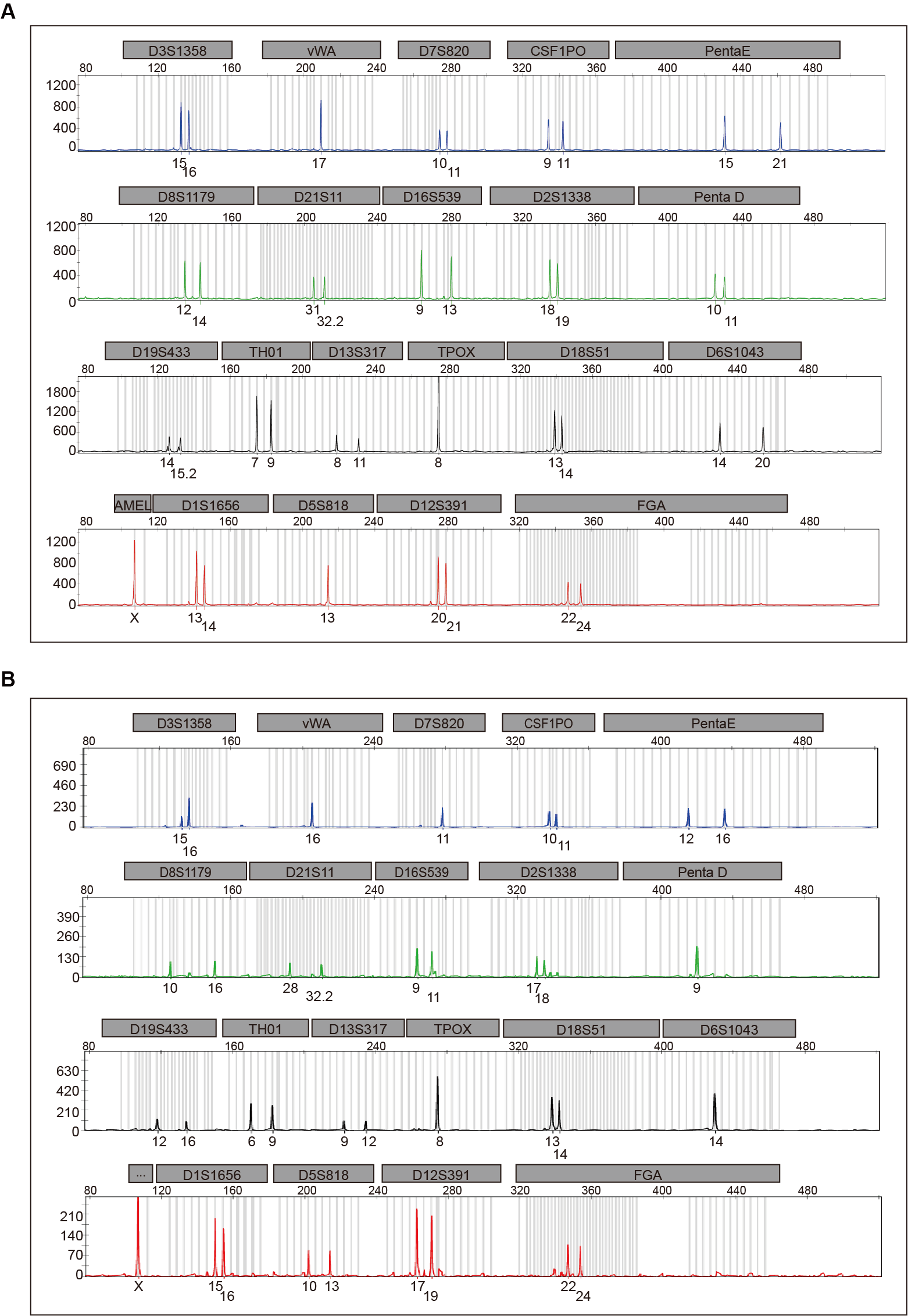


**Supplement figure 1. STR analysis of HES1 (A) and HES2 (B).**

Supplement: Supplementary file 1 — Supplementary Material 1. [file 13287_2025_4831_MOESM1_ESM.docx]
