# Supplementary material for: A simple and standardized method supports efficient derivation of clinical-grade human embryonic stem cells under feeder- and xeno-free conditions
Source: Stem Cell Res Ther. 2025 Dec 1;17:9. doi: 10.1186/s13287-025-04831-3 (PMC12772070; doi:10.1186/s13287-025-04831-3)
Supplement: Supplementary file 2 — Supplementary Material 2. [file 13287_2025_4831_MOESM2_ESM.docx]

**Table S1. GMP-grade reagents for hESC** **derivation, propagation and banking**

| **Reagent** | **Resource** | **Identifier** |
| --- | --- | --- |
| Thawing Media Kit | KITAZATO | VT102 |
| TeSR™-AOF Basal Medium | STEMCELL | 100-0402 |
| TeSR™-AOF 20X Supplement | STEMCELL | 100-0403 |
| Biolaminin 521 CTG | BioLamina | CT521-0501 |
| Y-27632 dihydrochloride | MCE | HY-10583G |
| BloodStor^®^ 100 | Biolife Solutions | 410301 |
| ReLeSR™ | STEMCELL | 100-0483 |
| CryoStor^®^ CS10 | Biolife Solutions | 210502 |

**Table S2. Antibody**

| **Reagent** | **Resource** | **Identifier** |
| --- | --- | --- |
| Rabbit monoclonal antibody to Nanog | HUABIO | Cat#ET1610-2; RRID: AB_3069903 |
| Mouse monoclonal antibody to Oct4 | HUABIO | Cat#HA601207; RRID: AB_3071926 |
| Mouse monoclonal antibody to OCT4 (OCT3) | Stem cell | Cat#60093;  RRID: AB_2801346 |
| Mouse monoclonal antibody to SSEA4 | Abcam | Cat#ab16287;  RRID: AB_778073 |
| Rabbit polyclonal antibody to PODXL (TRA-1-60) | Affinity | Cat#DF15469; |
| Rabbit monoclonal antibody to ATP1A1 | Abcam | Cat#ab76020;  RRID: AB_1310695 |
| Rabbit monoclonal antibody to ZO-1 | HUABIO | Cat#HA722797; RRID: AB_3675805 |
| Rabbit polyclonal antibody to cardiac troponin T (cTnT) | Abcam | Cat#ab45932;  RRID: AB_956386 |
| Mouse monoclonal antibody to sarcomeric alpha actinin (ACTN1) | Abcam | Cat#ab9465;  RRID: AB_307264 |
| Rabbit polyclonal antibody to FOXA2 | Millipore | Cat#07-633;  RRID: AB_390153 |
| Mouse polyclonal antibody to SOX17 | R&D Systems | Cat# MAB1924;  RRID: AB_2195646 |
| Mouse monoclonal antibody to NKX6.1 | DSHB | Cat#F55A12-C  RRID: AB_532379 |
| Rat monoclonal antibody to C-peptide Antibody | DSHB | Cat#GN-ID4  RRID: AB_2255626 |
| iFluor™ 594 conjugated goat anti-mouse IgG polyclonal antibody | HUABIO | Cat#HA1126;  RRID: AB_3675736 |
| iFluor™ 488 conjugated goat anti-rabbit IgG polyclonal antibody | HUABIO | Cat#HA1121;  RRID: AB_3675804 |
| Goat anti-mouse IgG H&L (Alexa Fluor^®^ 647) | Abcam | Cat#ab150115; RRID: AB_2687948 |
| Donkey anti-Rat IgG (H+L) Secondary Antibody, Alexa Fluor 488 | Life Technologies | Cat#A-21208;  RRID: AB_2535794 |
| Fluorescein (FITC) conjugated goat anti-rabbit IgG (H+L) | Proteintech | Cat#SA00003-2; RRID: AB_2890897 |

**Table S3. Reagents and kits**

| **Reagent** | **Resource** | **Identifier** |
| --- | --- | --- |
| Glutamax | Life | Cat#35050-061 |
| Glucose | Sigma | Cat#G7021 |
| Ascorbic acid | Sigma | Cat#A4544 |
| CHIR99021 | Selleck | Cat#S1263 |
| β-mercaptoethanol | Sigma | Cat#516732 |
| Wnt-C59 | Selleck | Cat#S7037 |
| SANT-1 | Selleck | Cat#S7092 |
| Retinoic acid | Sigma | Cat#R2625 |
| LDN193189 | Selleck | Cat#S7507 |
| Y27632 | Selleck | Cat#S1049 |
| TPB | Santa Cruz | Cat#SC-204424 |
| Nicotinamide | Sigma | Cat#N0636 |
| ALK5 inhibitor II | Enzo Life Sciences | Cat#ALX-270-445 |
| T3 | Sigma | Cat#T6397 |
| ISX9 | Selleck | Cat#S7914 |
| Heparin | Selleck | Cat#S1346 |
| γ-secretase inhibitor XXI | EMD Millipore | Cat#565789 |
| R428 | Selleck | Cat#S2841 |
| Zinc sulfate | Sigma | Cat#Z0251 |
| N-Acetyl-ʟ-cysteine | Sigma | Cat#A9165 |
| IWR-1 | Millipore | Cat#681669 |
| SB431542 | Selleck | Cat#1067 |
| Activin A | Stemimmune LLC | Cat#HST-A-1000 |
| KGF | Stemimmune LLC | Cat#HST-F7-0100 |
| EGF | Peprotech | Cat#AF-100-15 |
| bFGF | Stemimmune | Cat#HST-HS2-100 |
| Retinoic acid | MCE | Cat#HY-14649 |
| PDGF-BB | PeproTech | Cat#100-14B |
| DKK-2 | Peprotech | Cat#120-45 |
| Heregulin β-1 | MCE | Cat#HY-P7365 |
| IGF-1 | MCE | Cat#HY-P7018 |
| Accutase | Invitrogen | Cat#00-4555-56 |
| 20% knockout serum replacement | Gibco | Cat#10828028 |
| B27 | Gibco | Cat#12587010 |
| B27 supplement minus insulin | Gibco | Cat#A1895601 |
| B27 supplement with insulin | Gibco | Cat#17504044 |
| RPMI 1640 | Gibco | Cat#11875500BT |
| RPMI 1640 | Gibco | Cat#11879-020 |
| Matrigel | Corning | Cat#354277 |
| DMEM/F12 | Gibco | Cat#C11330500BT |
| mTESR1 | Stem Cell | Cat#85850 |
| MCDB 131 | Life | Cat#10372019 |
| DMEM | Gibco | Cat#C11965500CP |
| RNAiso plus | Takara | Cat#9109 |
| TB green^®^ premix ex taq™ | Takara | Cat#RR420A |
| PrimeScript RT reagent kit | Takara | Cat#RR047A |
| Alkaline phosphatase color development kit | Beyotime | Cat#C3250S |
| Cell Counting Kit-8 | Beyotime | Cat#C0038 |
| C-Peptide ELISA kit | Alpco | Cat#80-CPTHU-E01.1 |

**Table S4. Primers**

| **Name** | **Sequence** |
| --- | --- |
| NANOG-F | GAATAGCAATGGTGTGACGCAG |
| NANOG-R | GGGTGCACCAGGTCTGAGTGT |
| SOX2-F | AACCAGCGCATGGACAGTTA |
| SOX2-R | GACTTGACCACCGAACCCAT |
| SSEA3-F | CCCGCGCACGTTTCATC |
| SSEA3-R | AAGCCCAGTGGAGACTTTCG |
| LIN28-F | AGATCAAAAGGAGACAGGTGCT |
| LIN28-R | AGAATAGCCCCCACCCATTG |
| REX1-F | AACCATCGCTGAGCTGAAACAAA |
| REX1-R | ACCTCCAGGCAGTAGTGATCT |
| GAPDH-F | GGAGCGAGATCCCTCCAAAAT |
| GAPDH-R | GGCTGTTGTCATACTTCTCATGG |
| GDF3-F | TCTCCCAGACCAAGGTTTCTT |
| GDF3-R | CCTGAACCAGGAACAGAGCC |
| FGF4-F | AGCTCTATGGCTCGCCCTTC |
| FGF4-R | ATGCCGGGGTACTTGTAGGA |
| AQP1-F | CTGGGCATCGAGATCATCGG |
| AQP1-R | ATCCCACAGCCAGTGTAGTCA |
| ATP1A1-F | GGGAAGGGGGTTGGACG |
| ATP1A1-R | GCAGATGTTAATCCCCGGCT |
| TFAP2B-F | CACCTCCTAGAGACCAGGCT |
| TFAP2B-R | TCGTGCCGGTCCTCATAGATA |
| FOXC1-F | AGCAGCAGAACTTCCACTCG |
| FOXC1-R | AGTCGTAGACGAAAGCTCCG |
| ZEB1-F | TTACACCTTTGCATACAGAACCC |
| ZEB1-R | TTTACGATTACACCCAGACTGC |
| RARβ-F | TCTACACTGCGAGTCCGTCT |
| RARβ-R | TGATTGAGCAGTGTGCCGAT |
| RXRα-F | ATGGACACCAAACATTTCCTGC |
| RXRα-R | GGGAGCTGATGACCGAGAAAG |
| MEF2C-F | CATTGGCTACCCCAGTGGTT |
| MEF2C-R | TGAGTGCTAGTGCAAGCTCC |
| TBX5-F | CGTCTTTCCTGAGACTGCGT |
| TBX5-R | TGGGGACCACGGGATATTCT |
| GATA4-F | CGACACCCCAATCTCGTAG |
| GATA4-R | AGGCGTTGCACAGATAGTGA |
| RYR2-F | AAGATCATGCAGCCAGAGCC |
| RYR2-R | ACAACCAGGACGACTCCAAC |
| ACTN1-F | CTTTGACCGGGATCACTCCG |
| ACTN1-R | GGGGGTCGTTGCCAATATCA |
| cTNT-F | AGCGGAAAAGTGGGAAGAGG |
| cTNT-R | GGTCGAACTTCTCTGCCTCC |
| NKX2.5-F | CAAGGACCCTAGAGCCGAAA |
| NKX2.5-R | TGCGCCTGCGAGAAGAGCAC |
| Mycoplasma-F | GGGAGCAAACAGGATTAGATACCCT |
| Mycoplasma-R | TGCACCATCTGTCACTCTGTTAACCTC |

**Table S5. Compasion of the three methods for clinical-grade hESC derivation**

|  | **Souralova** **et al., 2023** | **Takada et al., 2022** | **This study** |
| --- | --- | --- | --- |
| Matrix | VTN-N/E-cadherin | iMatrix-511MG | Laminin-521 |
| Medium | NutriStem^@^ hPSC XF | StemFit AK03N | TeSR™-AOF |
| Y27632 (10 µM) | 3 days | Used but not specifically mentioned | 7 days |
| Total efficiency | 15.0% | 45.5% | 45.7% |
| The efficiency of different quality embryo (A/B/C) | Unclassified | Unclassified | Grade A: 100%  Grade B: 50%  Grade C: 28.6% |
